# 24-*O*-Ethylmanoalide, a Manoalide-related Sesterterpene from the Marine sponge *Luffariella* cf. *variabilis*

**DOI:** 10.3390/molecules13123184

**Published:** 2008-12-15

**Authors:** Anne Gauvin-Bialecki, Maurice Aknin, Jacqueline Smadja

**Affiliations:** Université de la Réunion, Laboratoire de Chimie des Substances Naturelles et des Sciences des Aliments, 97 715, Saint-Denis, La Réunion, France

**Keywords:** *Luffariella* cf. *variabilis*, Demospongiae, Manoalide-related sesterterpene, 24-*O*-ethylmanoalide

## Abstract

A new manoalide-related sesterterpene, 24-*O*-ethylmanoalide (**3**), was isolated from the Indian Ocean sponge *Luffariella* cf. *variabilis*, together with the known compounds manoalide (**1**), seco-manoalide, manoalide monoacetate and 24-*O*-methyl-manoalide (**2**). The structure of compound **3** was elucidated by interpretation of its spectroscopic data.

## Introduction

Marine sponges of the family Thorectidae (e.g. *Luffariella* [1,2,3,4,5,6,7,8,9,10,11,12,13,14,15], *Hyrtios* [16,17], *Thorectandra* [18], *Fasciospongia* [19,20,21,22,23], and *Aplynopsis* [24]) are known to be a rich source of novel bioactive sesterterpenoids. Some of them containing a γ-hydroxybutenolide moiety showed a strong anti-inflammatory activity. Manoalide (**1**), for example, the first sesterterpene to be reported from the Palauan sponge *Luffariella variabilis* by De Silva and Scheuer [1], has been extensively investigated as a potent inhibitor of phospholipase A_2_ (PLA_2_) [25,26,27,28,29,30,31,32,33]. Subsequently, many related metabolites with PLA_2_ inhibitory activity were reported [4,25,34,35,36,37,38,39]. In the course of our search for biologically active compounds from Indian Ocean marine organisms, our chemical investigation of a sponge from Mayotte Island belonging to the genus *Luffariella*, yielded manoalide (**1**) together with the known seco-manoalide [2], manoalide monoacetate [18], and 24-*O*-methylmanoalide (**2**) [13], as well as a new constituent which we have named 24-*O*-ethylmanoalide (**3**). In this paper, we describe the isolation and structure determination of compound **3**.

## Results and Discussion

The MeOH-CHCl_3_ extract of *Luffariella* cf. *variabilis* was subjected to solvent partitioning, as outlined in the Experimental section. The hexane fraction was repeatedly fractionated by silica gel column chromatography, followed by normal phase HPLC to afford manoalide monoacetate, 24-*O*-methylmanoalide (**2**) and 24-*O*-ethylmanoalide (**3**). The CCl_4_ and CHCl_3_ fractions were combined and chromatographed on a silica gel column to furnish manoalide (**1**) and seco-manoalide. The latter was further purified by normal phase HPLC. The known compounds manoalide (**1**), seco-manoalide, manoalide monoacetate and 24-*O*-methylmanoalide (**2**) were identified through comparison of their physical data (NMR and EIMS) with published information [1,2,3,13,16,18].

**Figure 1:**
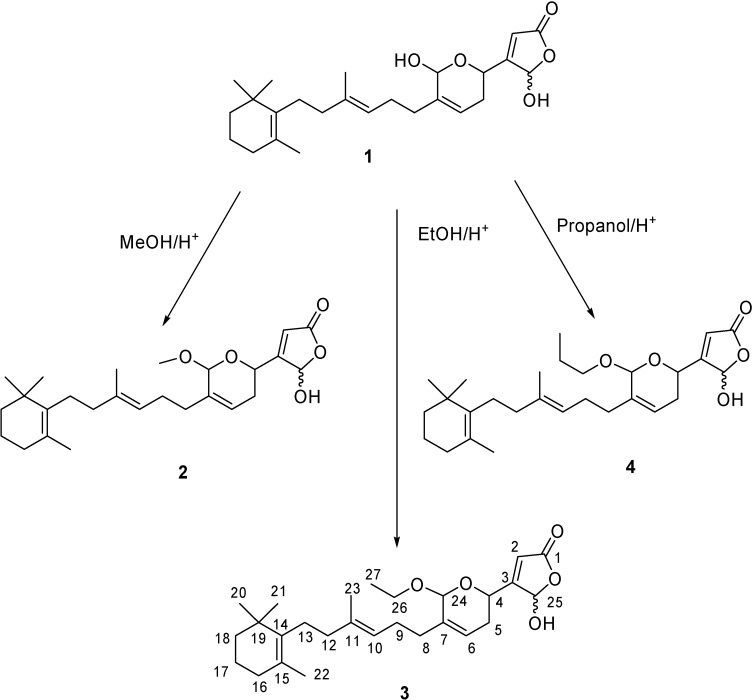
Possible chemical conversion of **1** into **2**, **3** and **4**.

Compound **3** was obtained as a colorless glass. The IR spectrum contained three bands at 3410, 1790 and 1762 cm^-1^, typical of a γ-hydroxybutenolide moiety, and a band at 1098 cm^-1^ supporting the presence of an ether group. The EIMS showed a molecular peak at *m/z* 444. This datum together with its ^1^H- and ^13^C-NMR spectra (Table 1) suggested the molecular formula C_27_H_40_O_5_. The mass spectrum showed an intense peak at *m/z* 137 and fragments ions at *m/z* 121, 107 and 95 derived from the *m/z* 137, implying the presence of the alkylated cyclohexenyl end group C_10_H_17_ commonly generated by manoalide-related sesterterpenes [18]. The ^1^H- and ^13^C-NMR of **3** were almost identical with those of manoalide (**1**). However, they showed the characteristic signals of an additional ethoxy group [*δ*_Η_ 3.55, 3.83 (2H, m, H-26), *δ*_Η_ 1.23, 1.24 (3H, t, *J* = 7.0 Hz, H-27), *δ*_C_ 64.0, 64.3 (C-26), and *δ*_C_ 15.3, 15.4 (C-27)]. The ether linkage between C-24 and C-26 was suggested by the ^13^C-NMR chemical shift of C-24 which resonated at a lower field (*δ*_C_ 97.1, 97.2) than the C-24 of (**1**) bearing an hydroxyl group (δ_C_ 91.2, 91.5). These data suggested structure **3** for 24-*O*-ethylmanoalide (Figure 1). Besides, pairs of two signals due to the same carbons or protons were detected in the ^1^H- and ^13^C-NMR spectra of **3** as similar to the signals of manoalide [16], which are ascribable to a mixture of stereoisomers. Compound **3** includes three asymmetric carbon atoms; C-4, C-24 and C-25. The axial nature of C-4 i.e. its *R*-configuration, was deduced from its coupling constants to the C-5 protons (10.5, 3.4 Hz) [1]. C-24 in **3** was also presumed to be an *R*-configuration. Indeed, the relative configuration between H-4 and H-24 was established to be *trans* on the basis of the similarity of chemical shifts of H-4, H-5, H-6 and H-24 in **3** with those of 24*R*-*O*-methylmanoalide and not 24*S*-*O*-methylmanoalide [13]. Therefore it was deduced that **3** is a mixture of C-25 epimers with *R*-configuration at C-4 and C-24.

It is interesting to note that compounds **2** and **3** may be suspected to be artifacts due to experimental procedure. Manoalide is indeed a hemiacetal and its extraction under some particular conditions - as shown in Figure 1 - would be expected to produce compounds **2** and **3**. If the conversion of **1** into **2** may be explained by the use of MeOH in the process of extraction [13], however the conversion of **1** into **3** requiring the use of EtOH/H^+^ remains unexplained. In the same way, in a previous report by Zhou and Molinski [14], manoalide (**1**) was presumed to be precursor of 24-*O*-propylmanoalide (**4**) (Figure 1), a manoalide derivative isolated from the Palauan sponge *Luffariella variabilis*.

**Table 1:** NMR Spectroscopic Data (CDCl_3_) for 24-*O*-ethylmanoalide (**3**)*^a^*.

position	δ_C_	δ_H_ (*J*, Hz)
1	170.3, 170.4	
2	117.5, 118.4	6.02, 6.19 s
3	167.4, 167.7	
4	62.3, 63.2	4.78, 4.86 dd (3.4, 10.5)
5	28.8, 29.1	2.20 m
6	120.6, 120.8	5.66 m
7	136.8, 137.1	
8	32.7	2.10 m
9	26.1	2.10 m
10	122.9	5.12 t (6.1)
11	137.1	
12	40.3	2.00 m
13	27.9	2.00 m
14	136.9	
15	127.1	
16	32.8	1.88 t (6.2)
17	19.6	1.53 m
18	39.9	1.39 m
19	35	
20	28.7	0.97 s
21	28.7	0.97 s
22	19.9	1.58 s
23	16.1	1.62 s
24	97.1, 97.2	4.89, 4.92 s
25	97.1, 97.7	6.09, 6.23 s
26	64.0, 64.3	3.55, 3.83 m
27	15.3, 15.4	1.23, 1.24 t (7.0)

^a ^Measured at 400 MHz (^1^H) and 100 MHz (^13^C).

However, according to the authors, the conditions of the process of extraction, partition and separation applied could not justify the conversion of **1** into **4**. Thus, on the basis of the above results, we suggest that 24-*O*-ethylmanoalide (**3**) and 24-*O*-propylmanoalide (**4**) be considered as “true” metabolites produced by a biosynthetic pathway, rather than artifacts arising from the isolation procedure.

## Experimental

### General

Optical rotations were measured on a Perkin-Elmer 341 polarimeter. IR spectra were determined on a Perkin-Elmer 1600 FT-IR spectrometer. ^1^H- (400 MHz) and ^13^C- (100 MHz) NMR spectra were recorded on a Brucker AMX-400, in CDCl_3_, with TMS as internal standard. Chemical shifts were reported in ppm and coupling constants (*J*) were reported in Hz. EI mass spectra were obtained on a Jeol AX-500 mass spectrometer. HPLC was performed on a Spectraseries P100 equipped with a differential refractometer (Thermoseparation products – Refractomonitor). A Merck Lichrospher Si-60 column (25 cm × 10 mm i.d.) was used.

### Animal material

The sponge *Luffariella* cf. *variabilis* (order Dictyoceratida, family Thorectidae) collected off Mayotte Island (Indian Ocean), in November 1995, was kept frozen until used. The material was identified by Dr N. Boury-Esnault (Station Marine d’Endoume – Marseille – France) and Pr P. Bergquist (School of Biological Sciences – Auckland – New Zealand). A voucher sample AGL-2-97M, has been deposited at the Laboratoire de Chimie des Substances Naturelles et des Sciences des Aliments (University of Reunion Island – France).

### Extraction and Isolation

Frozen sponge tissue (1,343 g dry weight after extraction) was cut up and homogenized in a Waring-blender in MeOH/CHCl_3_ (1:2). After filtration, the solvent was removed under reduced pressure to give the crude material (33.4 g), which was successively partitioned between equal volumes of aqueous MeOH, percentage adjusted to produced a biphasic solution, and a solvent series of *n*-hexane (yield 5.71 g), CCl_4_ (yield 11.95 g) and CHCl_3_ (yield 7.44 g). The remaining H_2_O soluble were extracted but did not contain any compounds of interest. A portion of the *n*-hexane fraction (2.98 g) was repeatedly subjected to silica gel columns using eluents of increasing polarity from 5% EtOAc in *n*-hexane to 10% EtOAc in *n*-hexane, to afford a mixture of manoalide monoacetate, 24-*O*-methyl-manaolide (**2**) and 24-*O*-ethylmanaolide (**3**). The resulting material was purified by semi-preparative HPLC over normal phase silica with hexane/EtOAc (7.5:2.5) to yield pure manoalide monoacetate (18 mg, 0.0026%, dry wt), **2** (13 mg, 0.0019%) and **3** (19 mg, 0.0027%). CCl_4_ and CHCl_3_ solubles were combined on the basis of TLC, and a 4.38 g portion was fractionated by silica gel column chromatography eluted with *n*-hexane/EtOAc using a step gradient of increasing EtOAc (9:1 to 7:3) to afford pure manaolide (**1**) (99 mg, 0.033%) and impure seco-manoalide. Final purification *via* HPLC using Si gel column with *n*-hexane/EtOAc (2:3) gave pure seco-manoalide (76 mg, 0.025%).

*24-O-ethylmanoalide* (**3**): colourless glass; [α]^25^_D_ + 63° (*c* 0.5, CHCl_3_); IR (CHCl_3_) ν_max_ 3410, 2925, 1790, 1762, 1098, 1040 cm^-1^; ^1^H- and ^13^C-NMR, see Table 1; EI mass spectrum *m/z* 444 [M] ^+^ (22), 426 (3), 398 (9), 380 (2), 261 (5), 203 (4), 177 (6), 137 (100), 123 (12), 121 (12), 107 (9), 95 (26), 81 (22).
